# Neonatal Thrombocytopenia and the Role of the Platelet Mass Index in Platelet Transfusion in the Neonatal Intensive Care Unit

**DOI:** 10.4274/balkanmedj.galenos.2020.2019.7.47

**Published:** 2020-04-10

**Authors:** Tuba Kasap, Şahin Takçı, Burcu Erdoğan Irak, Rüveyda Gümüşer, Ergün Sönmezgöz, Ali Gül, Osman Demir, Umut Safiye Şay Coşkun

**Affiliations:** 1 Department of Pediatrics, Tokat Gaziosmanpaşa University School of Medicine, Tokat, Turkey; 2 Department of Biostatistics, Tokat Gaziosmanpaşa University School of Medicine, Tokat, Turkey; 3 Department of Medical Microbiology, Tokat Gaziosmanpaşa University School of Medicine, Tokat, Turkey

**Keywords:** Intraventricular hemorrhage, neonatal intensive care unit, newborn, platelet mass index, platelet transfusion, thrombocytopenia

## Abstract

**Background:**

Neonatal thrombocytopenia is a common hematological abnormality that occurs in 20–35% of all newborns in the neonatal intensive care unit. Platelet transfusion is the only known treatment; however, it is the critical point to identify neonates who are really at risk of bleeding and benefit from platelet transfusion as it also has various potential harmful effects.

**Aims:**

To investigate the prevalence and risk factors of neonatal thrombocytopenia and its relationship to intraventricular hemorrhage in the neonatal intensive care unit and to determine whether the use of platelet mass index-based criteria could reduce the rate of platelet transfusion.

**Study Design:**

Retrospective cohort study.

**Methods:**

This study was conducted in the neonatal intensive care unit of a tertiary university hospital. The medical records of neonates in the neonatal intensive care unit with platelet counts <150×10^9^/L between January 2013 and July 2016 were analyzed.

**Results:**

During the study period, 2,667 patients were admitted to the neonatal intensive care unit, and 395 (14%) had thrombocytopenia during hospitalization. The rate of intraventricular hemorrhage was 7.3%. Multiple logistic regression analysis showed that although lower platelet counts were associated with a higher intraventricular hemorrhage rate, the effects of respiratory distress syndrome, sepsis, and patent ductus arteriosus were more prominent than the degree of thrombocytopenia. Thirty patients (7%) received platelet transfusion, and these patients showed a significantly higher mortality rate than their non-platelet transfusion counterparts (p<0.001). In addition, it was found that the use of platelet mass index-based criteria for platelet transfusion in our patients would reduce the rate of platelet transfusion by 9.5% (2/21).

**Conclusion:**

Neonatal thrombocytopenia is usually mild and often resolves without treatment. As platelet transfusion is associated with an increased mortality rate, its risks and benefits should be weighed carefully. The use of platelet mass index-based criteria may reduce platelet transfusion rates in the neonatal intensive care unit, but additional data from prospective studies are required.

Thrombocytopenia, which is defined as a platelet level below 150×10^9^/L, is a common hematological finding in newborns, occurring in 20–35% of all newborns admitted to a neonatal intensive care unit (NICU) (1,2). Neonatal thrombocytopenia (NT) may be mild to moderate or severe (platelet count <50×10^9^/L), and platelet transfusion (PT) may be required to avoid major bleeding. It is the critical point to identify and discriminate neonates who are really at risk of bleeding and benefit from PT as PT has also various potential harmful effects. Among these harmful effects, infectious, inflammatory, and hemodynamic risks are widely known (3,4,5,6,7). To decide properly, the risks and benefits of PT for each patient should be evaluated meticulously. In the last decade, the classical belief that thrombocytopenia causes major bleeding and death in neonates has somehow been replaced, and a more restrictive approach for PT has become common (7,8). There are various studies about PT in neonates, and finding measures for decreasing PT rate without an increase in the rates of bleeding and/or mortality is a popular topic. Using the criteria based on the platelet mass index (PMI) for PT is one of these approaches to avoid unnecessary PT, although there are some different results in the studies concerning these criteria (9,10,11,12,13). PMI is determined by multiplying platelet count by mean platelet volume and created from the knowledge that larger platelets are generally younger and qualitatively better and have better function in hemostasis than smaller ones (9). PMI was previously shown to be closely related to platelet function and a better indicator of the need for transfusion than platelet count alone, especially when the transfusion will be performed prophylactically (9).

In this study, we aimed to investigate the prevalence and risk factors of NT in the NICU of a tertiary university hospital and its relationship to intraventricular hemorrhage (IVH), which is among the most significant problems in neonates due to its association with a poor long-term prognosis (14,15). We also aimed to investigate whether the indications for PT based on platelet count were valid in transfused infants if criteria based on the PMI were considered.

## MATERIALS AND METHODS

This study was conducted retrospectively in the NICU of a tertiary university hospital. The medical records of neonates in the NICU with platelet counts <150×10^9^/L between January 2013 and July 2016 were analyzed. Thrombocytopenia was confirmed with peripheral blood smear, and patients with pseudothrombocytopenia were excluded.

The perinatal history, demographic features, age at onset of thrombocytopenia, lowest platelet count, transfusion requirements, time for resolution of thrombocytopenia, clinical features, and accompanying clinical conditions such as low Apgar score, intrauterine growth restriction (IUGR), sepsis, respiratory distress syndrome (RDS), patent ductus arteriosus (PDA), necrotizing enterocolitis (NEC, any stage), IVH, indirect hyperbilirubinemia (IHB), phototherapy treatment, and total parenteral nutrition (TPN) use were recorded from the medical files and blood bank records. For the low Apgar score, the 5^th^ minute Apgar score below 7 was considered (16). Placental insufficiency was diagnosed by clinical and sonographic findings. Perinatal hypoxia was diagnosed as Apgar score <5 at postnatal 5^th^ and 10^th^ minutes, and/or pH <7.00, or base excess more than -12 mmol/L in the umbilical cord blood and/or presence of brain damage consistent with neonatal hypoxic-ischemic encephalopathy in brain magnetic resonance imaging, and/or existence of multiorgan failure or damage (17). Sepsis was divided into two as confirmed and suspected. Confirmed sepsis was considered in infants who had at least two of the following criteria with positive blood culture: fever or hypothermia, tachypnea or apnea, tachycardia and abnormal white blood cells or increase in band/total neutrophils (18). “Suspected sepsis” was considered if the clinical and laboratory features were consistent with sepsis, but blood culture was negative. Other than this, sepsis was considered as “early onset” if it had occurred within the postnatal 3 days and “late onset” if later. RDS was diagnosed based on clinical, laboratory, and radiological findings. In this study, hemodynamically PDA was considered, and the diagnosis of PDA was made based on clinical and echocardiographic findings (ratio of left atrium diameter to aortic diameter >1.4) (19). For the diagnosis of NEC, Bell’s criteria were used, and any stage of NEC was recorded (20). Cranial ultrasonography (USG) was used to identify IVH, and the grade of hemorrhage was classified according to the Papile classification (21). As a routine protocol in our center, for preterm babies, cranial USG was performed once within the first 3 days and then 7^th^ and 21^st^ days of life and if required more frequently before discharge. Other than these routine days for preterms, cranial USG was also performed for term infants with thrombocytopenia.

Postmenstrual ages of all infants were within the neonatal time frame for the data “age at the onset of thrombocytopenia,” “lowest platelet day,” and “transfusion day.”

The patients were grouped according to the onset of thrombocytopenia. Thrombocytopenia before 72 h was defined as early-onset thrombocytopenia and after 72 h as late-onset thrombocytopenia (22).

To evaluate associated risk factors for severe thrombocytopenia, we divided the patients into the following categories based on their lowest platelet counts: mild, 100-150×10^9^/L; moderate, 50-100×10^9^/L; and severe thrombocytopenia, <50×10^9^/L.

The guidelines for administering PT in our NICU during this period were as follows: a platelet count of <20×10^9^/L if the infant is stable; a platelet count of 20-50×10^9^/L if the infant is unstable and/or had a birth weight of <1000 g and/or had previous major bleeding, and/or after an exchange transfusion and/or before a planned surgery, and/or a rapid decrease in the platelet level; a platelet count of 50-100×10^9^/L in an infant with active bleeding, and/or at the beginning of an exchange transfusion (23). PMI-based criteria were not considered in our NICU in the period that this study included, but we aimed to examine the indications for PT according to PMI-based criteria in platelet count-based transfused infants. PMI-based criteria recommends PT if PMI is <800 in pre/postoperative patients, <400 in unstable patients, and <160 in stable patients (24).

The complete blood counts were determined using a standard automated blood cell counter (CELL-DYN Ruby Hematology Analyzer; Abbott Laboratories, Chicago, IL) with ethylenediaminetetraacetic acid-anticoagulated blood samples.

The study was approved by the local institutional review board (18-KAEK-014).

### Statistical analysis

Statistical Package for the Social Sciences (SPSS) for Windows version 18.0 (SPSS, Inc., Chicago, IL) was used for all statistical analyses. The data are expressed as percentage, median, or mean ± standard deviation. Continuous variables were compared using the two-tailed t-test for parametrically distributed data or the Mann–Whitney U test for nonparametrically distributed data. Categorical variables were analyzed using the chi-square test. A multivariate logistic regression model was implemented to determine the relationships between the selected variables and IVH, and enter model was used. Variables that were significant in the univariate model and clinically thought to be effective on IVH were included in the model. The rate of IVH in thrombocytopenic neonates in NICU is reported as 4.5–33% (9,12). If the rate is considered as 4.5%, 388 children will be included in the study with 80% power, 5% type I error, and 0.0295 effect size. In all significant values, post-hoc power values were above 80%. In all analyses, p<0.05 was taken to indicate statistical significance.

## RESULTS

During the study period, 2,667 patients were admitted to the NICU. After excluding the patients with pseudothrombocytopenia (n=25), there were 395 patients (14%) with at least one recorded platelet count <150×10^9^/L. Of these thrombocytopenic neonates, 176 (44.6%) were girls, and 224 (58%) were preterm. The median birth weight was 2,322 g (410–5,900 g). The demographic characteristics, perinatal features, and postnatal event rates in the study population are given in Table 1.

The median age of the patients at the onset of thrombocytopenia was 2 days old (1–78 days). The median lowest platelet count was 101×10^9^/L (8–149×10^9^/L), and the median age at the lowest platelet count was 3 days (1–81 days). The mean and median values for postmenstrual ages of the infants at the onset of thrombocytopenia were 35.2±4.4 and 36.1 (22–44) weeks, respectively. Thrombocytopenia was present in 254 (64%) of the infants before they were 72 h old (early-onset thrombocytopenia) and in 141 (36%) after 72 h (late-onset thrombocytopenia). The median time for resolution of thrombocytopenia was 3 days (1–31 days). Comparison of early-onset and late-onset thrombocytopenia groups according to the demographic and clinical features revealed that the following factors were associated with late-onset thrombocytopenia: gestational age, birth weight, sepsis, IVH, PDA, NEC, IHB, phototherapy treatment, and TPN use (Table 2).

Of the thrombocytopenic neonates, 74 (18.7%) had severe thrombocytopenia, 119 (30%) had moderate thrombocytopenia, and 202 (51.3%) had mild thrombocytopenia. The rate of severe thrombocytopenia among all of the neonates in the NICU during the study period was 2.7%. To elucidate the risk factors for severe thrombocytopenia, the clinical features of these three groups were compared. The following factors were associated with severe thrombocytopenia: low Apgar score, IUGR, sepsis, IVH, PDA, RDS, TPN use, PT, and mortality (Table 3).

Among the 395 thrombocytopenic patients, 30 (7%) received PT. A comparison of these patients with those who did not receive PT is shown in Table 4. Twelve (40%) of the patients in the PT group died. The mortality rate was significantly higher in the PT group than in the non-PT group (p<0.001). The length of stay in the NICU was also higher in the PT group (p<0.001). As expected, the majority of the patients (29/30) who had PT were in the severe thrombocytopenia group (Table 4).

As thrombocyte transfusion was based on the platelet count, we also investigated whether these transfusions would have been indicated if PMI-based criteria had been considered. As these criteria are mostly for prophylactic PT, three patients who received transfusions because of active bleeding were excluded. The data of PMI were available for 21 of the remaining 27 patients with PT. These patients included the two who received transfusion based on the platelet count criteria, although no indications were present if PMI-based criteria were considered. Thus, for our patients, the use of PMI-based criteria would decrease the PT rate by 9.5% (2/21).

Twenty-nine of the total patient population had IVH; 6 infants had grade 4 IVH, 6 had grade 3, 3 had grade 2, and 14 had grade 1 IVH. As IVH is commonly encountered during neonatal care and is important in the long-term prognosis, we documented the relationships between patient characteristics, thrombocytopenia, and IVH. Significant associations were observed between IVH and gestational age, birth weight, age at onset of thrombocytopenia, lowest platelet count, lowest platelet count day, sepsis, PDA, RDS, and PT (Table 5). In this group, only three of the patients were term babies.

Multiple logistic regression analysis indicated that gestational age, lowest platelet count, sepsis, PDA, and RDS were associated with the development of IVH in thrombocytopenic neonates, with sepsis showing the greatest effect (Table 6).

## DISCUSSION

This study showed that although NT was encountered commonly in the NICU (14%), most cases were mild and resolved quickly without treatment (93%). Although lower platelet counts were associated with a higher IVH rate in the NICU, the effects of sepsis, RDS, and PDA were more prominent than the degree of thrombocytopenia. We also found that the use of PMI-based criteria for PT in neonates instead of platelet count-based criteria would decrease the PT rate by 9.5%.

The thrombocytopenia rate in the NICU has been reported to be 20–35%; this wide range is probably due to the differences between patient populations (1,2). The lower rate (14%) in our study may be explained by the heterogeneity of the patient population; in our center, the NICU includes both second-level and third-level NICU patients, which may have contributed to the relatively low rate of thrombocytopenia. The rate of severe thrombocytopenia in our study was 2.8%, which lies within the range of 2–25%, as reported in the literature (1,25).

In our study, 64% of the neonates exhibited thrombocytopenia within 72 h after birth as early-onset thrombocytopenia. In general, early-onset thrombocytopenia is usually mild and resolves without treatment (26). In the current study, probably due to the low number of transfusion cases, albeit not significantly so, the PT rate was lower in the early-onset group than in the late-onset thrombocytopenia group. Moreover, late-onset thrombocytopenia was associated with certain clinical conditions such as sepsis, PDA, NEC, and IVH. Sepsis and NEC are among the main causes of late-onset thrombocytopenia in neonates (22,27,28).

In the present study, IVH in neonates with thrombocytopenia was related to several factors, including gestational age, birth weight, time and severity of thrombocytopenia, and the presence of sepsis, PDA, and RDS. The significant association between thrombocytopenia severity and IVH seemed to be typical and a simple relation, but some studies have shown the opposite. For example, Baer et al. (28) focused exclusively on neonates in the NICU with thrombocyte counts <50×10^9^/L and found that IVH was not significantly related to the level of the lowest platelet count. Andrew et al. (29) concluded that maintaining a stable platelet count of >150×10^9^/L during the first week of life did not decrease the incidence of IVH. On the other hand, NT was found to be a risk factor for IVH in some studies, although there was no relationship between thrombocytopenia severity and IVH (1,25,30). However, Bolat et al. (31) reported a significant association between thrombocytopenia severity and IVH (grade ≥2), which is in line with our results. With regard to this association, it is not clear whether NT is a cause or a result of IVH, or whether both are coincidental results of some other neonatal issues, such as prematurity, low birth weight, or sepsis. In some studies of NT and IVH, the IVH was discovered before the onset of thrombocytopenia, implying that NT is not a direct cause (30,31,32). In a recent systematic review, which analyzed six studies, it was mentioned that there is insufficient evidence to show a causal relationship between platelet count and bleeding risk in neonates (33).

In this study, the PT rates among all of the NICU patients and the thrombocytopenic neonates in the NICU were 1.1% and 7%, respectively. However, rates of up to 10% for all NICU patients have been reported in the literature (34). This lower rate in our NICU may be multifactorial. First, strict criteria are applied for PT in our NICU to avoid unnecessary transfusions. In addition, our NICU includes both second-level and third-level NICU patients, which may have resulted in the lower rates of severe thrombocytopenia and, consequently, PT. In literature, there are various studies about the benefits and risks of PT in neonates. Andrew et al. (29) conducted a randomized controlled study of thrombocytopenic (50–150×10^9^/L) preterm infants and reported no difference in the incidence of bleeding between groups with and without PT; they concluded that prophylactic PT is not necessary (29). In another retrospective study comparing liberal and restricted PT approaches in very premature neonates, no significant difference in hemorrhagic events was found between the groups (35). Moreover, in our study, both the mortality and the IVH rate were significantly higher in patients who received PT. It is unclear whether this was due to the accompanying clinical conditions or directly to the transfusion process. In this regard, in some studies, authors have suggested that a higher mortality rate in transfused neonates was due to underlying illnesses that led to thrombocytopenia, while others have suggested that it was due to the transfusion itself (28,30,36). For example, Baer et al. (28) reported that the mortality rate increased when PT was performed at any platelet count and that this increment was proportional to the number of PTs administered. In a recently published multicenter prospective randomized trial in preterm neonates, differences regarding mortality or major hemorrhagic events between different thresholds (25 versus 50×10^9^/L) for PT in NICU were investigated, and it showed that the rates of both mortality and major hemorrhagic events were higher in the higher threshold group (7). In fact, this was not a new statement and had been suggested in some retrospective studies (28), but documentation of this in a prospective trial was striking. PT carries certain risks that are greater than those associated with the transfusion of other blood products, such as erythrocytes or plasma products, mainly due to the risk of bacterial infections. Given their preparation and storage rules, PT materials are prone to a greater risk of bacterial contamination (up to 10%), and this rate is too high to be ignored (3,4,5). Apart from this, as platelets release various inflammatory mediators and have important role also in inflammation other than hemostasis, it is thought that they could trigger or aggravate an inflammatory process that may contribute to increased mortality rate (6,7). It is also not fully understood which clear effects are observed in the transfused neonates with platelets, which are taken always from adults and indeed have some differences from the platelets of the neonates (37,38). Other than these, PT has some volume-related hemodynamic risks, which may be critical for the neonates especially for the preterm newborns since they have some other common cardiovascular problems accompanying and are very sensitive and fragile in terms of vascular structures in the brain and also other organs (6,7). Because of all these risks, more thought should be put into the process of deciding to perform PT in a neonate.

Although the number of patients in the transfused group in our study was small, our observations indicated that the rate of transfusions may be further decreased by 9.5% using PMI-based criteria. Since the 1970s, it has been known that larger platelets function better in plug formation, and PMI-based criteria were established because of the idea that bleeding risk is not related solely to platelet count but also platelet size and function (10,39,40). Previous reports have also suggested that the use of PMI-based criteria may be a good strategy for reducing unnecessary transfusions in the NICU (9,10). In a prospective study, Gerday et al. (9) reported that the use of these criteria led to a decrease in the PT rate with no associated increase in bleeding. Kahvecioglu et al. (11), in a retrospective study that was similar to our study, reported that the use of PMI-based criteria led to a decrease of 11% in the PT rate. However, some studies have shown no difference in the PT rates associated with the use of the two sets of transfusion criteria. For example, in a prospective randomized study, Zisk et al. (12) observed no differences between the two groups in terms of transfusions, mortality, or hemorrhagic problems. Yavuzcan et al. (13) reported similar results for PT rates with the use of two sets of criteria. The results of all these limited number studies about PMI-based criteria for PT in neonates may suggest that the use of these criteria, at least with no increase in bleeding rates, may be beneficial for decreasing PT rates in the NICU and deserves additional investigation.

This study has some limitations, mainly due to its retrospective nature. For example, we did not know the exact diagnoses and etiologies of NT in all of the patients. On the other hand, a relatively large sample size is the strength of the study. Also, this study is among the rare studies in literature regarding the use of PMI-based criteria in thrombocytopenic neonates. Prospective studies are needed, and these will provide more accurate information.

Based on the results of this study, we conclude that NT was usually mild and often resolved without treatment. Moreover, PT was associated with a higher mortality and IVH rate. Considering these findings, the risks and benefits should be weighed carefully before administering PT to neonates in the NICU. Prospective studies regarding the use of PMI-based criteria to decrease PT rates are required.

## Figures and Tables

**Table 1:**
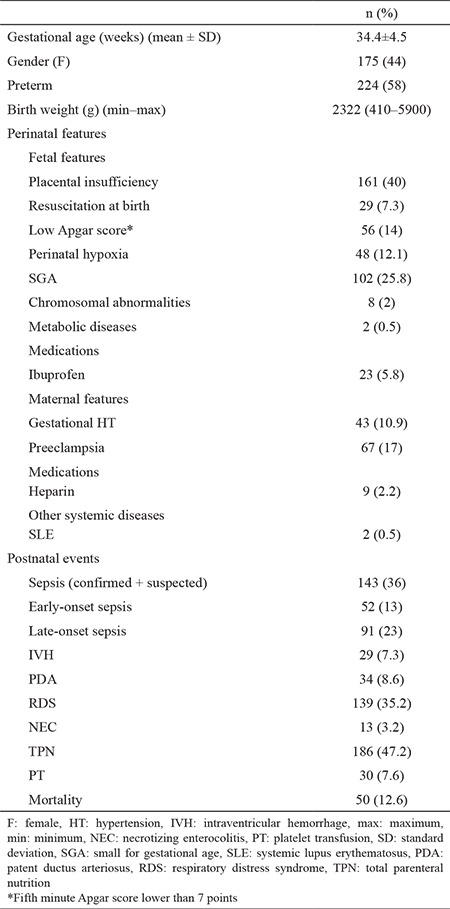
Demographic characteristics, perinatal features, and postnatal events in the study

**Table 2:**
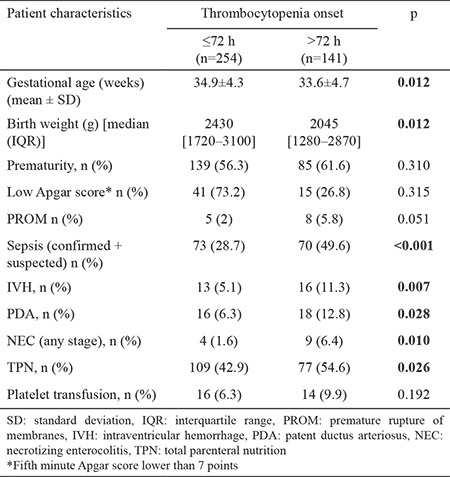
Comparison of the patient characteristics with regard to early-onset and late-onset thrombocytopenia

**Table 3:**
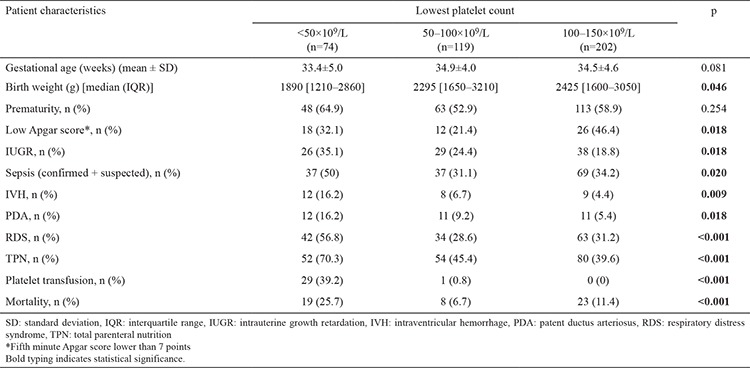
Comparison of the patient groups based on the thrombocytopenia severity

**Table 4:**
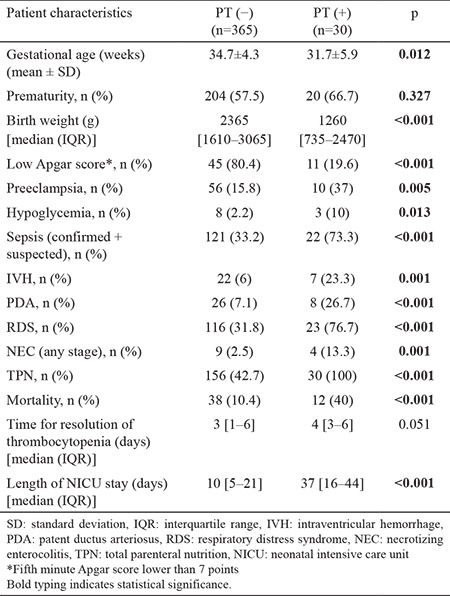
Comparison of the patient characteristics based on the need for a platelet transfusion.

**Table 5:**
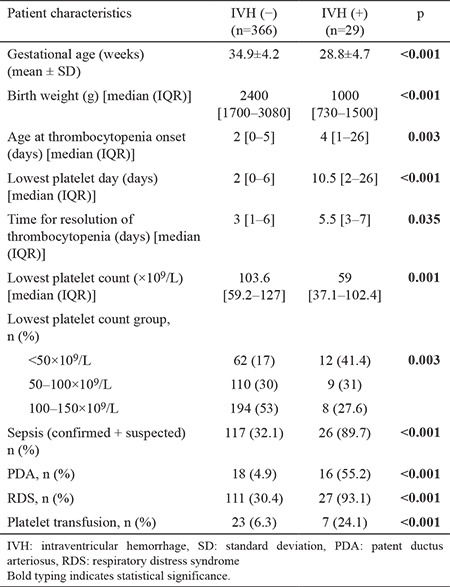
Comparison of thrombocytopenic neonates with and without IVH.

**Table 6:**
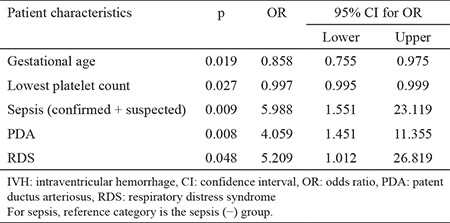
Results of the IVH multiple logistic regression model
